# The subcellular distribution of phosphorylated Y‐box‐binding protein‐1 at S102 in colorectal cancer patients, stratified by *KRAS* mutational status and clinicopathological features

**DOI:** 10.1002/1878-0261.70060

**Published:** 2025-05-26

**Authors:** Konstanze Lettau, Stephan Forchhammer, Birgit Fehrenbacher, Lejla Mahmutovic, Marcus Scharpf, Gunnar Blumenstock, Martin Schaller, Irina Bonzheim, Shayan Khozooei, Mahmoud Toulany

**Affiliations:** ^1^ Department of Radiation Oncology University Hospital Tuebingen Tuebingen Germany; ^2^ Department of Internal Medicine I University Hospital Tuebingen Tuebingen Germany; ^3^ Department of Dermatology University Hospital Tuebingen Tuebingen Germany; ^4^ Institute of Pathology and Neuropathology University Hospital Tuebingen Tuebingen Germany; ^5^ Department of Clinical Epidemiology and Biostatistics University Hospital Tuebingen Tuebingen Germany

**Keywords:** colorectal cancer, KRAS mutation, prognosis, YB‐1 phosphorylation

## Abstract

Oncoprotein Y‐box‐binding protein‐1 (YB‐1) is involved in all cancer hallmarks. One of the most studied post‐translational modifications of YB‐1 is phosphorylation on Serine 102 (S102), which is involved in cancer progression. *KRAS* mutations are frequent, have been associated with poor prognosis and therapy resistance, and they are considered a major stimulator of S102 YB‐1 *in vitro*. In this study, a relationship between S102 YB‐1 phosphorylation in subcellular fractions and *KRAS* mutation was investigated in CRC tissues, and its association with clinicopathological parameters was analyzed. Immunohistochemistry on 36 patient samples and 5 normal tissue samples highlighted nuclear S102 YB‐1 was specific to cancer tissues. Nuclear S102 YB‐1 was expressed in 47.2% of tumor tissues, which was positively correlated with KRAS mutation (*P* = 0.017). There was no significant association between cytoplasmic S102 YB‐1 with KRAS mutation status (*P* = 0.391). Further studies in larger cohorts are needed to validate the observed results. The significant association between S102 YB‐1 in the nucleus and *KRAS* mutation may suggest YB‐1 as an effective target to improve survival of CRC patients with KRAS‐mutated tumors.

## Graphical abstract

This study identifies nuclear YB‐1 S102 phosphorylation as a marker associated with *KRAS* and *FBXW7* mutations in colorectal cancer. Mutated KRAS correlates specifically with nuclear, not cytoplasmic, S102 YB‐1. These findings provide the first *ex vivo* evidence of this link in CRC and suggest future studies should assess the prognostic and therapeutic relevance of its subcellular localization.

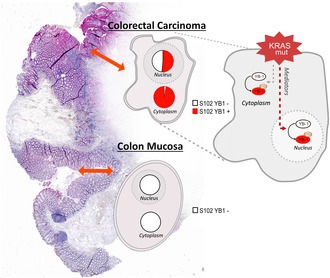

**Abbreviations**
5‐FU: 5‐fluorouracil
AKT: AKT serine/threonine kinase
BRAF: B‐Raf Proto‐Oncogene, Serine/Threonine Kinase
CRC: colorectal cancer
CRT: chemoradiotherapy
CT: chemotherapy
EGFR: epidermal growth factor receptor
ERK: extracellular signal‐regulated kinase
FBXW7: F‐box and WD repeat domain‐containing 7
FFPE: formalin‐fixed, paraffin‐embedded
FOLFIRI: 5‐fluorouracil + leucovorin + irinotecan (chemotherapy regimen)
G2/G3: Tumor Grade 2 (moderately differentiated)/Grade 3 (poorly differentiated)
HE: hematoxylin and eosin
IHC: immunohistochemistry
KRAS: Kirsten Rat Sarcoma Viral Oncogene Homolog
LVI: lymphovascular invasion
M: metastasis (distant metastasis staging component)
MAPK: mitogen‐activated protein kinase
MDR1/ABCB1: multi‐drug resistance protein 1 /ATP binding cassette subfamily B member 1
MSI: microsatellite instability
MSS: microsatellite stable
MVP: major vault protein
N: node (lymph node involvement staging component)
NGS: next‐generation sequencing
NLS: nuclear localization signal
OS: overall survival
PI3K: phosphatidylinositol‐3 kinase
PIK3CA: phosphatidylinositol‐4,5‐bisphosphate 3‐kinase catalytic subunit alpha
Pn: perineural invasion
PTM: post‐translational modification
RSK: ribosomal S6 kinase
S102: serine at position 102 (phosphorylation site)
T: tumor (primary tumor staging component)
TNM: tumor‐node‐metastasis (cancer staging system)
TOP‐1: topoisomerase 1
TP53: tumor protein p53
UICC: Union for International Cancer Control
V: vascular invasion
VEGF: vascular endothelial growth factor
YB‐1: Y‐box binding protein 1

## 1 Introduction

Colorectal cancer (CRC) is the third most diagnosed cancer and the fourth leading cause of cancer‐related deaths worldwide. Molecular biomarkers are specific for genetic or biochemical changes that can be detected in a patient's cancer cells or tissue and are used to help with diagnosis, classification, and prediction of the behavior of cancers.

The *KRAS* oncogene is a common molecular biomarker, which is mutated in approximately 45% of CRC [1] and has been associated with resistance to certain treatments [2, 3]. KRAS, through multiple signaling cascades among them the MAPK/ERK and PI3K/AKT pathways, mediates tumor cell proliferation, survival, DNA repair, and migration in CRC [4]. Molecular biomarkers that have been identified in CRC include epidermal growth factor receptor (EGFR), the vascular endothelial growth factor (VEGF), and the microsatellite instability (MSI) status, which is an indicator of DNA repair mechanisms in cancer cells. Additional prognostic and predictive markers of CRC are *TP53, PIK3CA*, and *BRAF* mutations [2]. Based on those molecular markers defined thus far, current treatment involves multimodal approaches, such as surgery and (neo‐) adjuvant chemoradiotherapy (CRT), chemotherapy (CT), and molecular targeted therapy. CT regimens often contain cytotoxic agents combined with molecular targeting approaches, *e.g*., anti‐EGFR or anti‐VEGF molecular targeted therapies depending on *KRAS, BRAF, NRAS*, and *PIK3CA* mutation status. MSI and *BRAF* ^*V600E*^ mutated tumors are frequently treated with checkpoint inhibitors and/or tyrosine kinase inhibitors.

Y‐box binding protein 1 (YB‐1) is a multifunctional oncoprotein involved in the development and maintenance of various cancer entities, including but not limited to breast, colorectal, and lung cancers [5, 6, 7, 8]. Under certain conditions, YB‐1 undergoes multiple post‐translational modifications (PTMs) that can affect its stability, transcriptional and translational activities, and subcellular localization. The phosphorylation of YB‐1 at S102 plays a critical role in its binding affinity to DNA and RNA, impacting the regulation of genes crucial in cancer progression [9, 10].

Studies have demonstrated that YB‐1 expression promotes the expression of multiple oncogenes in CRC by regulating the transcription of key genes involved in cell cycle progression, angiogenesis, and metastasis [11]. Moreover, YB‐1 affects DNA damage repair mechanisms, potentially contributing to resistance against CRT by upregulating DNA repair genes [10, 12, 13]. Nuclear YB‐1 also binds to the multi‐drug‐resistance receptor 1 (MDR1)‐promoter upon genotoxic stress (*i.e*., chemotherapy treatment) and thus it is involved in therapy resistance *in vitro* [14]. Additionally, YB‐1 can directly interact with some DNA repair proteins, including MSH2, Ku80, WRN, and DNA polymerase delta, following cisplatin‐induced DNA damage [15].

YB‐1 phosphorylation at S102 is mainly regulated by the mitogen‐activated protein kinase (MAPK)/extracellular signal‐regulated kinase (ERK) and phosphatidylinositol‐3 kinase (PI3K)/AKT pathways. This phosphorylation can be activated either after stimulation of the components of upstream cascades through external factors, or it can be constitutively phosphorylated by mutations of upstream components like KRAS [11]. It has been demonstrated that *KRAS* mutation leads to constant YB‐1 phosphorylation at S102 in CRC cells [13]. This was shown to be mediated via the p90 ribosomal S6 kinase (RSK) and AKT [10, 13, 16]. An effective inhibition of YB‐1 phosphorylation at S102 was achieved by co‐targeting RSK and AKT [13, 16]. As mentioned earlier, PTMs affect YB‐1 translocation to the nucleus. In this context, YB‐1 can be translocated to the nucleus under certain conditions, including hypoxia [17], cisplatin treatment [18], and UV irradiation [19]. However, accumulating evidence indicates that following ionizing irradiation (IR), phosphorylation of nuclear YB‐1 at S102 occurs in the nucleus via the translocation of its kinase, *i.e*., RSK [20].

In line with the function of YB‐1 in DNA repair, an association between total YB‐1 expression and poor prognosis has been observed in patients with colorectal, hepatocellular, lung, breast, prostatic, renal, and bladder cancers, as well as melanomas and osteosarcomas [21, 22, 23, 24, 25, 26, 27, 28, 29, 30, 31, 32, 33]. YB‐1 is reported to be strongly expressed in CRC in comparison to normal colon tissues [34, 35, 36]. Shiraiwa et al. [33] reported a reduced overall and recurrence‐free survival in CRC patients, which showed high expression of YB‐1 in the nucleus in over 60% of the tested stage three CRC patient samples. The phosphorylation status of YB‐1 was not evaluated in that study.

Thus far, only two other studies have specifically investigated phosphorylated YB‐1 at S102 in cancer specimens. One study focused on breast cancers and noted increased S102 YB‐1 in relapsed and therapy resistant tumors [37]. Another study by Kosnopfel et al. [32] focused on melanoma, showing that S102 phosphorylation of YB‐1 increased nuclear localization *in vitro*. Interestingly, increased unphosphorylated cytoplasmic YB‐1 seemed to increase EMT and invasiveness of melanoma cells in comparison to S102 phosphorylated YB‐1.

Given the prognostic value of YB‐1 expression, no studies have evaluated the association between YB‐1 phosphorylation status and *KRAS* mutation in CRC patients. In the current study, we investigated the subcellular localization of S102 YB‐1 in regard to its correlation with therapeutically relevant genetic mutations, *i.e*., mutation in *KRAS* and *BRAF* in association with other clinical factors. The data obtained demonstrate that S102‐phosphorylated YB‐1 is present in most CRC specimens in the cytoplasm independent of *KRAS* mutation status. However, *KRAS* mutation and not *BRAF* mutation leads to the nuclear accumulation of YB‐1 phosphorylation.

*KRAS* mutations are associated with resistance to EGFR inhibitors and other targeted therapies in CRC [3]. Identifying S102 YB‐1 as a downstream effector in this tumor entity in human specimens offers a potential indirect targeting strategy for *KRAS*‐mutant tumors, circumventing the inherent challenges of directly inhibiting mutant KRAS itself. It provides a novel molecular insight into KRAS‐driven tumorigenesis, identifies a potential biomarker, and opens up new therapeutic avenues. This finding addresses critical gaps in our understanding of KRAS signaling in patients and may lead to improved outcomes for patients with this challenging molecular subtype of CRC.

## 2 Materials and methods

### 2.1 Ethics

Patient samples were taken in accordance with broad consent given by the patients; therefore, the experiments were undertaken with written consent of each subject. The study presented was in accordance with and beforehand permitted by the ethics committee (525/2020BO) of the university Tuebingen. The experiments were performed in accordance with German legislation and the Declaration of Helsinki.

### 2.2 Patient samples and study population

Formalin‐fixed, paraffin‐embedded (FFPE) patient samples from CRC patients were obtained from the biobank of the Medical Faculty University Tuebingen. The samples were collected from patients who underwent surgery at the university clinic Tuebingen between 2019 and 2021. Thirty‐six tumor samples (13 male, 23 female) and 5 normal tissue samples were included in this study. The corresponding clinical and pathological data were obtained from the clinic archive. Mean patient age of onset was 69. Most samples turned out to be from patients with UICC Stage III/IV, with a broad range regarding TNM staging, which was available for 35 patients (Table 1).

**Table 1:** (A, B) Characteristics of the study population and (C) observed mutation frequencies.

A			
		Absolute	Percent
	Mean age of onset	69	
Gender	Male	13	36.1%
	Female	23	63.9%
	Total	36	100.00%
UICC stage	I	1	2.9%
	II	5	14.3%
	III	11	31.4%
	IV	18	51.4%
T	1	0	0.0%
	2	1	2.9%
	3	19	54.3%
	4	15	42.9%
N	0	8	22.9%
	1	13	37.1%
	2	14	40.0%
M	0	11	36.7%
	1	19	63.3%
B			
	Localisation	Absolute	
	Coecum	10	
	Colon ascendens	13	
	Colon transversum	1	
	Colon descendens	2	
	Sigmoid/Rectosigmoid	8	
	Rectum	2	
C			
	Mutation frequency (%)		Reported reference (%)
*TP53*	56.8		58.6
*KRAS*	40.5		35–45
	G12C	6	3.4
	G12D	40	36
	G12V	26	21.8
	G13D	13	18.8
	Q61K	13	5.0
	Total	100	
*BRAF*	24.3		3.6–10
*SMAD4*	13.5		9.8
*ERBB2*	5.4		9.4
*MET*	2.7		3
*FBXW7*	10.8		10–11
*CTNNB1*	8.1		4.7
*PIK3CA*	13.5		17.6
*AKT1*	2.7		0
MS‐Deregulation	21.6		13

### 2.3 DNA extraction and next‐generation sequencing

Genomic DNA was extracted using the Maxwell^®^ RSC DNA FFPE Kit and the Maxwell^®^ RSC Blood Kit, and the Maxwell^®^ RSC instrument (Promega, Madison, WI, USA) according to the manufacturer's instructions.

Targeted multigen mutation screening was performed by Next‐Generation Sequencing (Ion GeneStudio S5, Thermo Fisher Scientific, Waltham, MA, USA) using the AmpliSeq Colon and Lung Cancer Panel v2 (hotspot regions in 22 genes: *KRAS, EGFR, BRAF, PIK3CA, AKT1, ERBB2, PTEN, NRAS, STK11, MAP2K1, ALK, DDR2, CTNNB1, MET, TP53, SMAD4, FBXW7, FGFR3, NOTCH1, ERBB4, FGFR1, FGFR2*). Amplicon library preparation and semiconductor sequencing were done according to the manufacturers' manuals using the Ion AmpliSeq Library Kit v2.0, the Ion Library TaqMan Quantitation Kit on the LightCycler 480 (Roche, Basel, Switzerland), the Ion 510 & Ion 520 & Ion 530 Kit—Chef on the Ion Chef, and the Ion 540 Kit‐Chef (Thermo Fisher Scientific). Output files were generated with Torrent Suite 5.16. Variant calling of non‐synonymous somatic variants compared to the human reference sequence hg19 was performed using Ion Reporter Software (Thermo Fisher Scientific, version 5.18). Detection thresholds were set at an allele frequency of 5%. Variants called by the Ion Reporter Software were visualized using the Integrative Genomics Viewer (IGV; Broad Institute, Cambridge, MA, USA; Version 2.11.9) to exclude panel‐specific artifacts. The NCBI dbSNP database (including GnomAD, ExAC, and TOPMED) was used to exclude SNPs.

### 2.4 Immunostaining

For the immunofluorescence staining, paraffin sections were deparaffinized and rehydrated. Slides were incubated in EDTA buffer pH 9.0 (Zytomed, Berlin, Germany) using a steam cooker. Sections were blocked using donkey serum (Sigma‐Aldrich, #F9663, St. Louis, MO, USA) and incubated with the following antibodies: mouse anti‐YB‐1 (Abcam, #ab268094, Cambridge, United Kingdom) and rabbit anti‐P‐YB‐1 (Cell Signaling, #2900, Leiden, the Netherlands). Bound antibodies were visualized using donkey anti‐mouse‐Alexa‐647 (Dianova, #715‐606‐151, Hamburg, Germany) and donkey anti‐rabbit‐Cy3 (Dianova, #711–166‐152). YO‐PRO‐1 (Thermo Fisher Scientific, #Y3603) was used for nuclear staining. Sections were analyzed using an LSM 800 confocal laser scanning microscope (Zeiss, Oberkochen, Germany). Images were processed with the software ZEN 2.3 (blue edition).

To conduct Immunohistochemistry staining, after fixation in 4% formalin, the tissue was dehydrated, embedded in paraffin, cut into 3 μm sections, deparaffinized, and stained with hematoxylin and eosin (HE). Immunohistochemistry (IHC) was performed with rabbit anti‐P‐YB‐1 S102 (Cell Signaling, #2900) on an automated immunostainer (Leica Bond‐MAX; Leica Biosystems, Wetzlar, Germany) according to the company's protocols. The slides were subsequently digitalized using a digital slide scanner (NanoZoomer 2.0‐HT; Hamamatsu Photonics K.K., Hamamatsu, Japan) and analyzed using NDP.view2. To stratify the staining intensity of YB‐1 phosphorylation at S102, the stained slides were analyzed by two experts and classified into four groups based on the intensity of the staining.

### 2.5 Statistical analysis

Data was analyzed using IBM SPSS Statistics 28 software. Categorical data are reported as numbers and percentages; continuous data are summarized with the mean or with the median in skewed distributions. To describe relationships between two categorical variables, cross‐tabulation was used. Dealing with limited sample sizes, Fisher's exact test of independence was performed for 2 × 2 table analysis. To compare paired proportions, McNemar's test was used. Graphs and tables were generated using IBM SPSS Statistics, Microsoft Excel, and Serif Affinity Designer.

## 3 Results

### 3.1 Stratifying the staining intensity of YB‐1 phosphorylation at S102

**Fig. 1:**
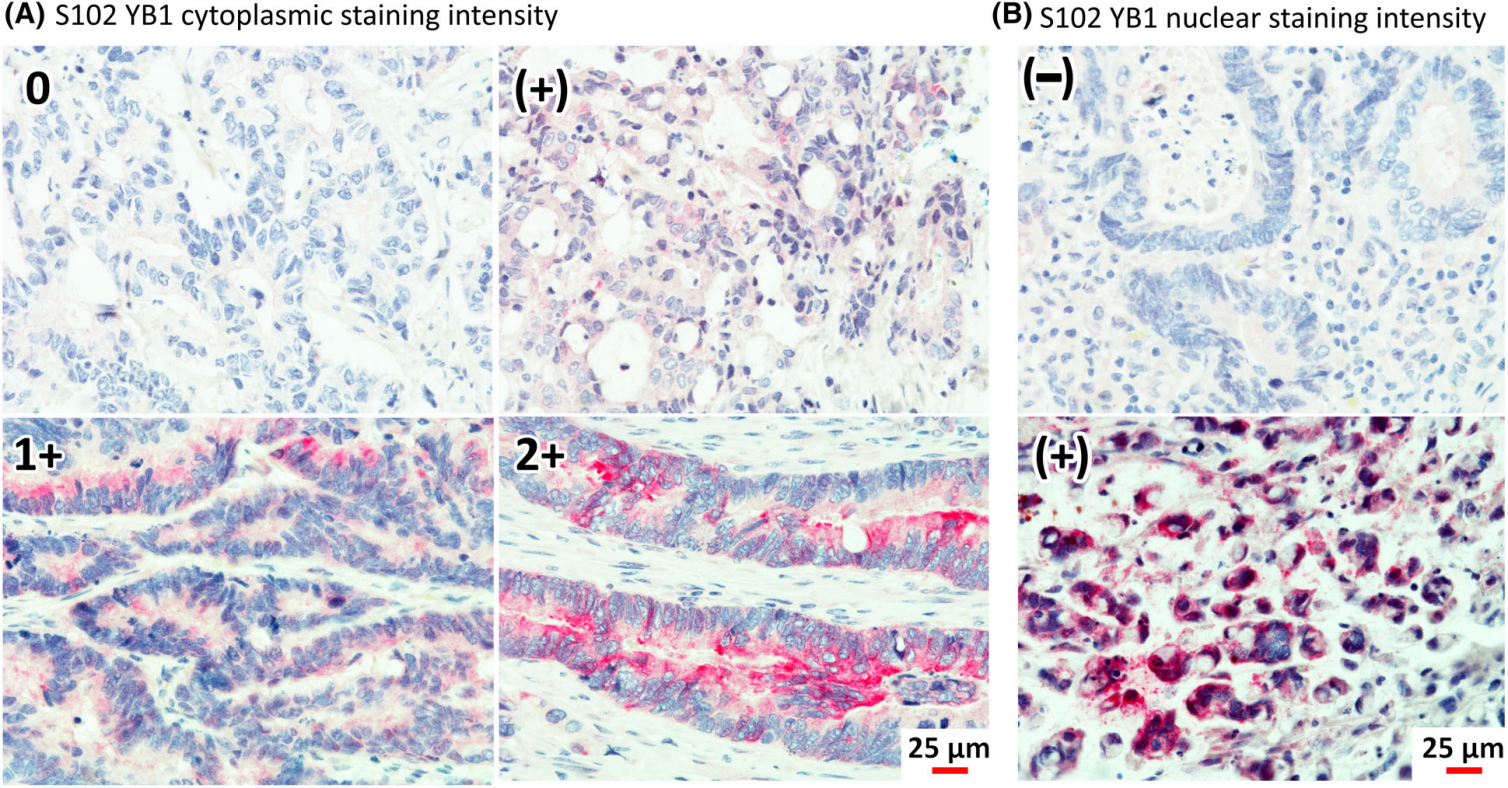
Demonstration of S102 YB‐1 immunohistochemistry staining intensity in the semiquantitative system in (A) cytoplasm (0 (*n* = 1), (+) (*n* = 14), 1+ (*n* = 11), 2+ (*n* = 10)) and (B) nucleus (− (*n* = 19), + (*n* = 17)). Scale bar 25 μm. Original magnification ×400.

**Fig. 2:**
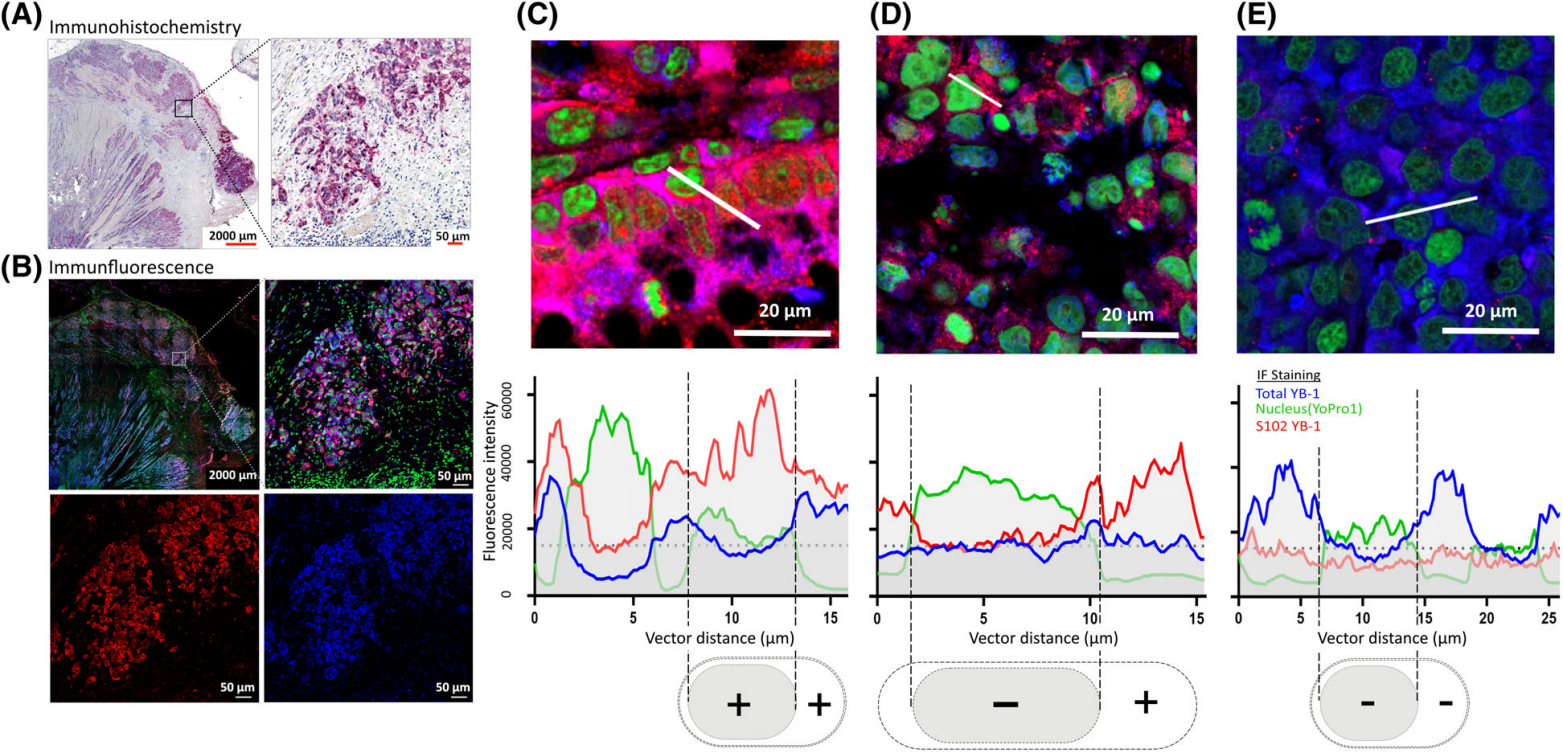
Subcellular staining of YB‐1 phosphorylation in CRC patient tissues (*n* = 36). (A, B) Immunohistochemistry and immunofluorescence staining. Original magnification ×50 (top left images). (C) Negative staining of phospho‐YB‐1 in the cytoplasm and in the nucleus (*n* = 1). (D) Positive staining of S012 YB‐1 (P‐YB‐1) in the cytoplasm but not in the nucleus (*n* = 18). (E) Positive staining of P‐YB1 both in the cytoplasm and nucleus (*n* = 17). Blue = AF647 (total YB‐1), Red = Cy3 (S102 P‐YB1), Green = YoPro1 (nucleus).

S102 YB‐1 immunohistochemistry staining intensity was semiquantified in the nucleus and cytoplasm (Fig. 1). The YB‐1 phosphorylation at S102 staining in the cytoplasm was scored as (0) = no stain, (+) = <25% of cells with S102, (1+) = <75% of cells with S102, (2+) = >75% of cells with S102 in tumor regions (Fig. 1A). In addition, nuclear staining of S102 YB‐1 was also subgrouped (Fig. 1B). In combined immunohistochemistry and immunofluorescence stains, tumor areas show a high signal for YB‐1 and S102 YB‐1 (Fig. 2A,B) and on a subcellular level, S102 YB‐1 signal can be discriminated from total YB‐1 signal (Fig. 2C–E). Based on microscopy, three forms of cancer cells could be identified: high S102 YB‐1 signal in the cytoplasm and nucleus simultaneously, high S102 YB‐1 only in the cytoplasm, and overall low S102 YB‐1 signal (Fig. 2C–E).

### 3.2 Mutation analysis of the studied samples

The cohort characteristics are summarized in Table 1A–C. The study cohort showed mostly higher staged cancers (UICC III and IV), and most specimens were right‐sided CRC and sigmoid CRC (Table 1A,B). To study the impact of *KRAS* mutation on YB‐1 phosphorylation at S102, next‐generation sequencing (NGS) was performed. Besides *KRAS* mutation, the mutation status of genes that have been reported to be frequently mutated in CRC was analyzed (Table 1C). Among the genes analyzed, *TP53* was the most frequently mutated gene observed, followed by *KRAS* mutations in 40.5% of samples. Mutation in *KRAS* was present in 44% of right colon samples and 42% of left colon samples. Among the observed *KRAS* mutations, the most frequent mutations were in codons 12 and 13 of exon 2 (G12/G13) within 87% of all *KRAS*‐mutated samples. Mutation of *KRAS* in codon 61 (Q61C) was detected in 2 samples. *BRAF* mutations, mutually exclusive with *KRAS* mutations, were found in 24.3% of samples. *F‐box and WD repeat domain‐containing 7 (FBXW7*) mutations were detected in about 10% of CRC samples. The frequency of all other gene mutations detected by the used panel is summarized in Table 1C.

### 3.3 Subcellular distribution of phosphorylated YB‐1

**Fig. 3:**
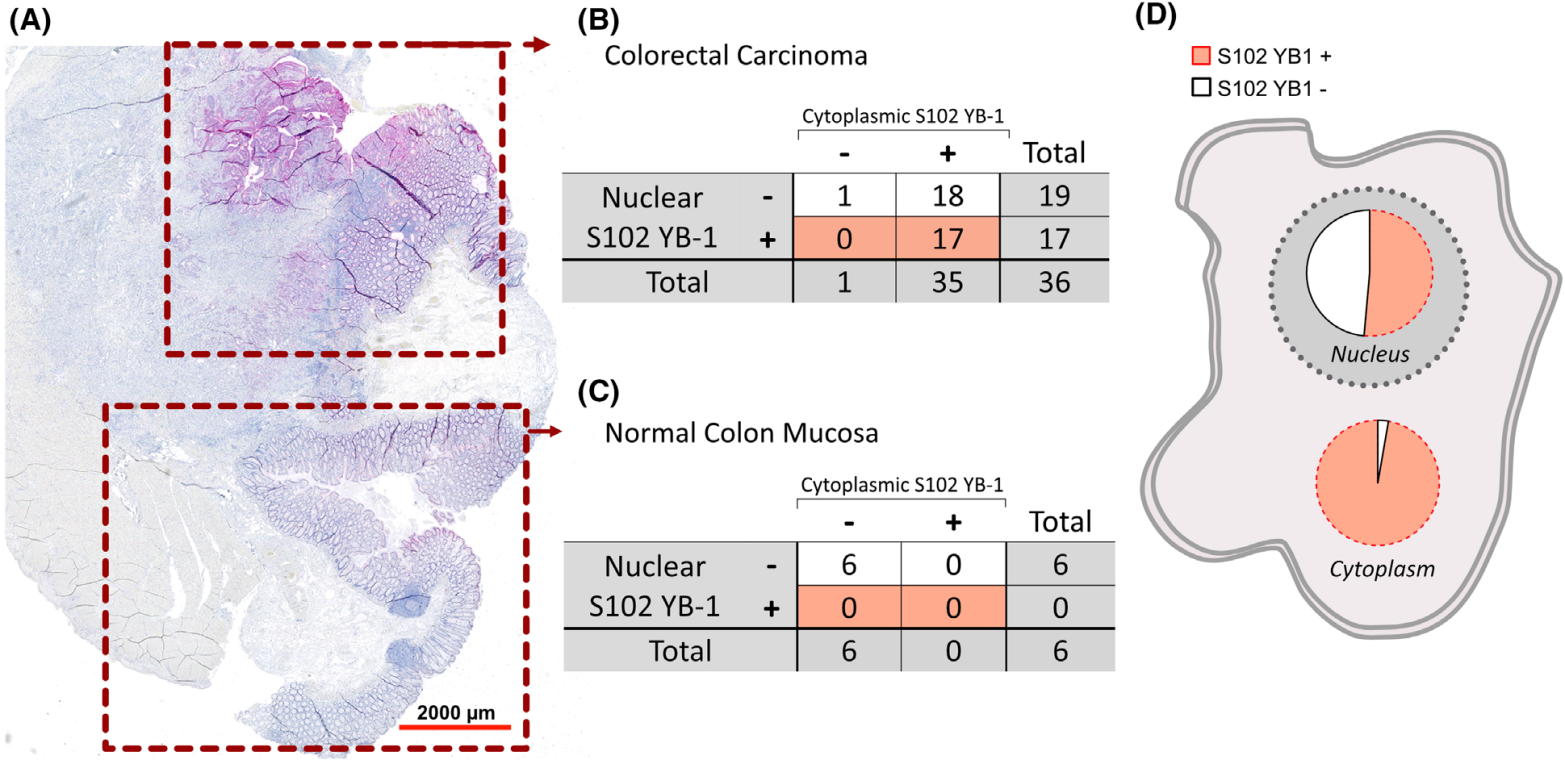
The staining of YB‐1 phosphorylation in CRC patient tissues. (A) Representative IHC staining of the staining intensity of phosphorylated YB‐1 at S102 in normal mucosa vs. adenocarcinoma of the same patient. (B, C) Summarizing cross tables showing the expression of S102 YB‐1 in the cytoplasm vs. nucleus in cancerous (B) and in normal tissues (C) included in this study. (D) Relative representation of S102 YB‐1‐negative and ‐positive fractions of samples.

Carcinomatous areas in the samples presented strong staining intensity of S102 phospho‐YB‐1 (Fig. 3A,B). In contrast, in most samples, areas with normal mucosa were negative for S102 phosphorylation of YB‐1 (exemplarily shown in Fig. 3C). In line with this observation, none of the included normal tissue samples (*n* = 6) showed S102 YB‐1 phosphorylation in the cytoplasm or nuclei. Nuclear S102 YB‐1 was only present in samples that presented S102 YB‐1 in the cytoplasm. In total, 1 tumor sample and 6 normal tissue samples were negative for S102 YB‐1 in the cytoplasm, of which all were also negative for S102 YB‐1 in the nucleus. Among the CRC samples analyzed, 47.2% presented YB‐1 phosphorylation in the nucleus and in the cytoplasm, simultaneously (Fig. 3D).

### 3.4 Associations between S102 YB‐1 levels and clinicopathological and molecular pathological parameters of CRC patients

**Fig. 4:**
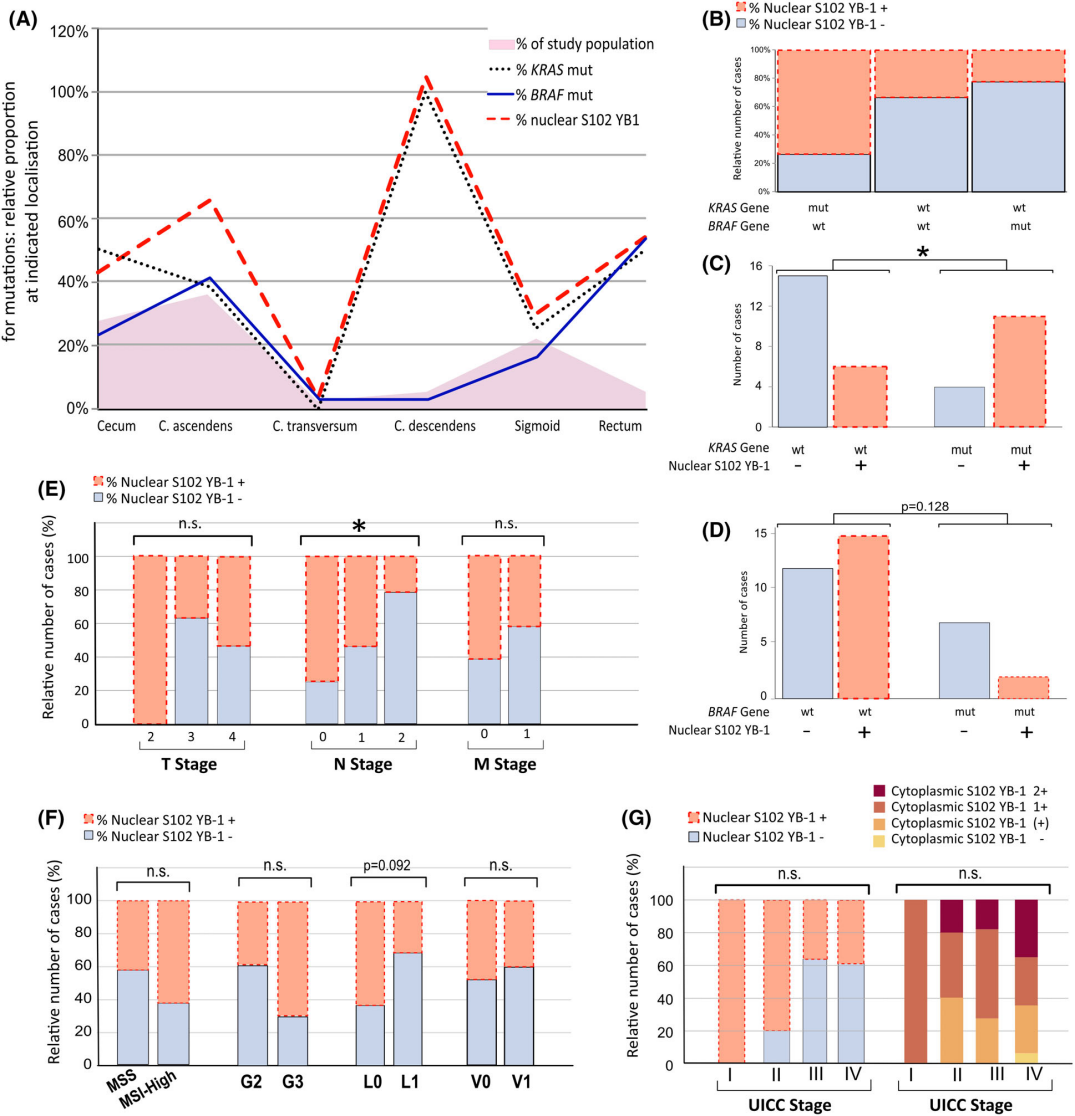
Associations between S102 YB‐1 levels and *KRAS/BRAF* mutations, as well as additional clinicopathological parameters, in CRC patients. (A) Distribution of CRC localization in the study population (gray area). Relative proportion of *KRAS/BRAF* mutation and nuclear P‐YB‐1 at indicated locations in the colon (*n* = 36). (B) Bar graph showing the relative number of samples with nuclear P‐YB‐1 dependent on *KRAS/BRAF* mutation status (*KRAS* mut *n* = 15, wt *n* = 12, *BRAS* mut *n* = 9). (C, D) Bar graph showing the absolute number of cases with and without nuclear P‐YB‐1, dependent on *KRAS* mutation status (C) and *BRAF* mutation status (D) (*n* = 36). (E, F) Impact of TNM staging, microsatellite status, grading, and lymphangio/vascular invasion on the phosphorylation status of YB‐1. Bar graphs show the proportion of samples with nuclear P‐YB1 in the T2–4 stage (E), N0, N1, and N2 subgroups (E), M0 and M1 subgroups, MSS and MSI‐High subgroups, G2 and G3 subgroups (F), L0 and L1 subgroups, V0 and V1 subgroups (F) on the intensity of phosphorylated YB‐1 in the nucleus. (G) Bar graph showing the proportions of S102 YB‐1 in the nucleus and the cytoplasm in UICC stages I–IV (G) (*n* = 36). mut, mutated; n.s., non‐significant; wt, wildtype. For statistical analysis (B–G), Fisher's exact test was applied.

The distribution of CRC localization in the study population is presented in Fig. 4A. Among the distributions, *KRAS* mutation in association with S102 phosphorylated YB‐1 was primarily observed in tumors localized in the descending colon, sigmoid, and rectum (Fig. 4A). In tumors from these areas, nuclear S102 YB‐1‐positive status is significantly linked to *KRAS*‐mutated status (*P* = 0.017) (Fig. 4B,C). *KRAS* mutation led to the enhanced phosphorylation of nuclear YB‐1 at S102. As depicted in Fig. 4A, the presence of S102 YB‐1 in the nucleus and *KRAS* mutation closely overlaps. Most interestingly, there was a stronger coincidence of mutated *KRAS* and YB‐1 phosphorylation in the left colon (colon descendent, sigmoid, rectum) in which 60% of tumors were *KRAS* mutated (Fig. 4A). Among them, 80% presented high values of S102 YB‐1 (*P* = 0.072 for association). *KRAS* mutation status was not associated with enhanced phosphorylation of YB‐1 in the cytoplasmic fraction (Table 2). The study population also showed that – while *KRAS* and *BRAF* mutations were mutually exclusive – the probability of nuclear S102 YB‐1 was reduced in *BRAF‐*mutated samples compared to the wildtype samples with a non‐significant negative association (*P* = 0.128) (Fig. 4D). No association was observed between *TP53, PIK3Ca*, and *SMAD4* mutations and nuclear phosphorylation of YB‐1. Interestingly, *FBXW7* mutation correlated significantly with the nuclear presence of S102 YB‐1 (*P* = 0.04) (Table 2).

**Table 2:** Characteristics of the study population stratified by nuclear and cytoplasmic S102. Study population gender, primary CRC location, TNM stage (T = Tumor; N = Node; M = Metastasis), as well as histopathological markers for lymphovascular invasion (LVI), vascular invasion (V) and perineural invasion (Pn), as well as UICC stage. Selected panel genes analyzed in the Archer pan‐tumor assay, as well as microsatellite status stratified by presence of S102 in the nucleus and cytoplasm. Layer row percentages are calculated to depict distribution among samples. Median age of tumor onset, disease‐free survival (DFS) and overall survival (OS) are depicted with corresponding 95% upper and lower confidence interval (CI). *P*‐values as calculated by Fisher's Exact 2‐sided test are graphed next to the analyzed parameters. Significant associations are marked in dark green. Mut, mutated; n.a., not available; Wt, wildtype.

		Nuclear S102 YB‐1			Cytoplasmic S102 YB‐1		
		Low	High		Low	High	
		Layer row *N* %	Layer row *N* %	*P*‐value	Layer row *N* %	Layer row *N* %	*P*‐value
Gender	Male	61.5%	38.5%	0.502	0.0%	100.0%	1.000
	Female	47.8%	52.2%		4.3%	95.7%	
Location	Right colon	47.8%	52.2%	0.725	4.3%	95.7%	1.000
	Left colon	58.3%	41.7%		0.0%	100.0%	
T Stage	2	0.0%	100.0%	0.386	0.0%	100.0%	1.000
	3	63.2%	36.8%		5.3%	94.7%	
	4	46.7%	53.3%		0.0%	100.0%	
N Stage	0	25.0%	75.0%	0.043	0.0%	100.0%	0.600
	1	46.2%	53.8%		7.7%	92.3%	
	2	78.6%	21.4%		0.0%	100.0%	
M Stage	0	38.5%	61.5%	0.477	0.0%	100.0%	1.000
	1	57.9%	42.1%		5.3%	94.7%	
Metastasis site	Non‐hepatic distant metastasis	60.0%	40.0%	0.539	20.0%	80.0%	0.179
	Hepatic metastasis	54.5%	45.5%		0.0%	100.0%	
	No metatasis	33.3%	66.7%		0.0%	100.0%	
Grading	2	61.5%	38.5%	0.092	3.8%	96.2%	1.000
	3	30.0%	70.0%		0.0%	100.0%	
LVI	0	38.9%	61.1%	0.092	0.0%	100.0%	0.486
	1	70.6%	29.4%		5.9%	94.1%	
V	0	52.0%	48.0%	0.723	4.0%	96.0%	1.000
	1	60.0%	40.0%		0.0%	100.0%	
Pn	0	50.0%	50.0%	0.487	3.1%	96.9%	1.000
	1	100.0%	0.0%		0.0%	100.0%	
UICC Stage	1	0.0%	100.0%	0.214	0.0%	100.0%	1.000
	2	20.0%	80.0%		0.0%	100.0%	
	3	63.6%	36.4%		0.0%	100.0%	
	4	61.1%	38.9%		5.6%	94.4%	
*KRAS*	wt	71.4%	28.6%	0.017	4.8%	95.2%	1.000
	mut	26.7%	73.3%		0.0%	100.0%	
*BRAF*	wt	44.4%	55.6%	0.128	3.7%	96.3%	1.000
	mut	77.8%	22.2%		0.0%	100.0%	
*P53*	wt	46.7%	53.3%	0.736	0.0%	100.0%	1.000
	mut	57.1%	42.9%		4.8%	95.2%	
*PIK3CA*	wt	51.6%	48.4%	1.000	3.2%	96.8%	1.000
	mut	60.0%	40.0%		0.0%	100.0%	
*SMAD4*	wt	54.8%	45.2%	0.650	3.2%	96.8%	1.000
	mut	40.0%	60.0%		0.0%	100.0%	
*FBXW7*	wt	59.4%	40.6%	0.040	3.1%	96.9%	1.000
	mut	0.0%	100.0%		0.0%	100.0%	
Microsatellite	Stable (MSS)	57.7%	42.3%	0.429	3.8%	96.2%	1.000
	Instable (MSI)	37.5%	62.5%		0.0%	100.0%	
*ERBB2*	wt	55.9%	44.1%	0.216	2.9%	97.1%	1.000
	mut	0.0%	100.0%		0.0%	100.0%	
*MET*	wt	54.3%	45.7%	0.472	2.9%	97.1%	1.000
	mut	0.0%	100.0%		0.0%	100.0%	
*FGFR1*	wt	54.3%	45.7%	0.472	2.9%	97.1%	1.000
	mut	0.0%	1 00.0%		0.0%	100.0%	
*CTNNB1*	wt	51.5%	48.5%	1.000	3.0%	97.0%	1.000
	mut	66.7%	33.3%		0.0%	100.0%	
*AKT1*	wt	51.4%	48.6%	1.000	2.9%	97.1%	1.000
	mut	100.0%	0.0%		0.0%	100.0%	
		Median (CI)	Median (CI)		Median (CI)	Median (CI)	
Age of CRC onset (years)		76 (68–80)	65 (60–76)	0.075	n.a.	71 (67–79)	n.a.
DFS (months)		7 (5–26)	5 (0–13)	0.700	n.a.	6 (0–13)	n.a.
OS (months)		19 (16–42)	28 (19–44)	0.116	n.a.	26 (18–42)	n.a.

In total, no significant correlation was found between UICC stage and cytoplasmic or nuclear S102 YB‐1; however, it has to be noticed that nuclear S102 YB‐1 seemed to be less present in higher UICC stages (III/IV) (Fig. 4G, Table 2).

Moreover, we investigated the impact of microsatellite status, lymphovascular invasion (LVI), TNM staging, and grading on the phosphorylation status of nuclear YB‐1 (T = Tumor; N = Node; M = Metastasis). Compared to microsatellite stable tumors (MSS, *n* = 28), CRC with high microsatellite instability (MSI‐High, *n* = 8) was found to have a higher probability of expressing nuclear S102 YB‐1 (Fig. 4F, Table 2); however, this was not statistically significant. There was a negative trend between nuclear S102 YB‐1 and LVI (non‐significant, *P* = 0.092) (Fig. 4E, Table 2). A trend was also observed between S102 YB‐1 in the nucleus and the younger age of tumor onset (non‐significant, *P* = 0.075) (Table 2). No significant association was observed with either T‐staging or grading. However, it was noted that G3 tumors were more likely to express S102 YB‐1 in the cytoplasm than G2 tumors, even though this was not statistically significant (*P* = 0.092) (Fig. 4F, Table 2). There was no association between vascular invasion (V), perineural invasion (Pn), and general metastasis (M‐Stage) with YB‐1 phosphorylation at S102 (Fig. 4E,F, Table 2). There was a significant reduction in the proportion of patients with lymph node metastasis (*P* = 0.043) when S102 YB1 was present in the nucleus (Fig. 4E, Table 2). No correlation was found with liver metastasis (Table 2).

## 4 Discussion

It is widely assumed that YB‐1 needs to be activated via phosphorylation to mediate its oncogenic effects on the cancer hallmarks, explicitly in colorectal cancers [13]. It is assumed that YB‐1 transfers to the nucleus when it is not phosphorylated, and that phosphorylation then leads to cytoplasmic or nuclear retention. In both compartments, ribosomal s6 kinase (RSK) is involved in S102 phosphorylation of YB‐1 [20, 38]. In this study, nuclear S102 YB‐1 was detected in 47.2% of samples, while cytoplasmic S102 YB‐1 was observed in all samples except one.

While YB‐1 primarily resides in the cytoplasm, its involvement in transcription and translation regulation allows it to be found in both compartments [39]. Under specific conditions such as stress response and drug resistance, YB‐1 can translocate to the nucleus [39]. Notably, PTMs play a crucial role in regulating YB‐1 translocation to the nucleus by inducing conformational changes that might expose the nuclear localization signal (NLS) sequences. In *in vitro* studies, *KRAS* mutation has been shown to increase the phosphorylation of YB‐1 at S102 in breast cancer and CRC [10, 13, 20] without inducing YB‐1 translocation to the nucleus [20]. Here we examined the phosphorylation status of YB‐1 at S102 in the cytoplasm and nucleus differentially *ex vivo* in CRC tissues from 36 patients and the impact of genetic mutations on the subcellular localization. The general mutation frequency of the observed genes was comparable to that published previously [1, 40, 41].

This study could demonstrate that nuclear S102 YB‐1 was significantly associated with *KRAS* mutation status, but not correlated with *BRAF* mutation status. An additional significant correlation was observed between the nuclear presence of S102 YB‐1 and *FBXW7* mutation.

Our study did not show a significant association between S102 YB‐1 and tumor stage and grading, even though nuclear S102 YB‐1 was more frequent in higher grade tumors. Other studies have noted the correlation of nuclear and cytoplasmic YB‐1 expression with neoplasia of higher grade in Head and Neck cancer [42] and CRC [36].

No strong associations could be found between clinicopathological parameters such as MSI‐H and UICC stage. The study at hand did not see any significant association between nuclear S102 YB‐1 and M‐Stage. Intriguingly, our study found a significant negative correlation between nuclear S102 and lymphonodal metastasis (N‐Stage). Interestingly, Kosnopfel et al. [32] demonstrated in melanoma specimens that YB‐1 increases the invasion and metastasis only in its S102‐ unphosphorylated form. There are no other studies evaluating explicitly phosphorylated YB‐1 in metastatic spread and thus far, main conclusions were only drawn for total YB‐1 expression. Previous studies in CRC patients have suggested that YB‐1 expression might be associated with lymphovascular invasion and lymph node metastasis [29]. A similar study in gastric cancer has demonstrated that YB‐1 expression is significantly associated with lymph node spread [43]. In contrast, Ardito et al. [44] examined 66 CRC Patients and found no significant correlation between the total YB‐1 expression and clinical prognostic factors, such as N‐stage. The role of S102 YB‐1 in lymphatic spread is not clear and further studies are needed to elaborate on this subject. YB‐1's involvement in modulating genes related to metastasis and its role in promoting angiogenesis could play a role in this association. However, these modulators are not yet known.

Not much is reported on the correlation of YB‐1 and *BRAF* mutations. It is generally assumed that YB‐1 is phosphorylated via AKT and RSK [13, 16]. RSK is a downstream kinase of BRAF, so activating mutations of BRAF should mechanistically increase S102 YB‐1. However, it seems to be not as straightforward. The study at hand is the first to evaluate this relationship *ex vivo* and found that S102 YB‐1 does not correlate with *BRAF* mutation in CRC in the same manner as *KRAS* mutation. The role of BRAF in CRC strongly differs from other tumor entities, *i.e*., monotargeting of BRAF is not effective in CRC and requires additional EGFR targeting to assert anti‐tumor effects [45, 46].

The study at hand is the first to find a link between S102 YB‐1 and *FBXW7* mutations. In CRC, mutations in FBXW7, a tumor suppressor, are involved in oncogenic signaling pathways [47]. FBXW7 targets various proteins for ubiquitin‐mediated degradation, and *FBXW7* mutations induce mTOR (mechanistic target of rapamycin) activity, and activation by the mTOR complex can influence YB‐1 phosphorylation [48, 49].

Ardito et al. [44] demonstrated that cytoplasmic YB‐1 expression was present in all CRC cases and strong YB‐1 expression was the only independent risk factor for predicting an increased recurrence of CRC. Current clinical studies that investigated the impact of YB‐1 expression on therapy outcome in breast, cervical, head and neck cancer, as well as sarcoma patients, identified nuclear YB‐1 expression as a negative prognostic factor for OS, disease‐free survival, or progression‐free survival [22, 42, 50, 51, 52]. We recommend stratification by *BRAF* wildtype in larger cohorts for future evaluation of the prognostic role of S102 YB‐1, as *BRAF* mutation was determined to be one of the main prognostically relevant mutations in CRC [53, 54]. Also, based on the observation that nearly all CRC samples in this study were positive for S102 YB‐1, we recommend nuclear S102 YB‐1 as a more differentiated marker for further survival analysis in larger cohorts. Due to the limited sample size, survival analysis was not performed in this study.

The following segments of the discussion will revolve around the role of *KRAS* mutation and consecutive YB‐1 S102 phosphorylation in chemotherapy response. In general, YB‐1 has been shown to promote the expression of genes involved in chemotherapy resistance, including the DNA repair genes such as *XRCC1, BRCA1* [55] and the MDR gene *ABCB1*, which codes for the P‐glycoprotein [14, 56, 57]. Additionally, in CRC cells, it was demonstrated that YB‐1 mediates the expression of the major vault protein (MVP), which is also suspected to be responsible for MDR in cancers [58, 59].

In this context, 5‐Fluorouracil (5‐FU), which is a main part of the first‐line chemotherapy regimen in CRC, induces YB‐1 binding to the MVP promoter potentially enhancing MDR even in cells without mutation‐driven YB‐1 expression [57]. Blocking YB‐1 phosphorylation via the targeting of upstream kinases leads to increased sensitivity to 5‐FU in CRC cells [13]. Additionally, a study by Tsofack et al. [60] has demonstrated that the overexpression of YB‐1 leads to oxaliplatin resistance in human CRC cell lines.

We can therefore conclude from *in vitro* experiments that CRC with high levels of phosphorylated YB‐1 are at risk of therapy resistance to 5‐FU and oxaliplatin, and that blocking YB‐1 phosphorylation may be a promising therapeutic strategy.

Irinotecan, which is crucial in CRC treatment and often part of initial therapy regimens (*i.e*., FOLFIRI). The efficacy of topoisomerase 1 (TOP‐1) inhibitors has demonstrated a higher selectivity toward *KRAS‐*mutated CRC cells [61]. Furthermore, it has been shown that YB‐1 interacts with TOP‐1 and enhances its activity [62, 63]. This complex formation leads to increased sensitivity to the TOP‐1 inhibitor, camptothecin [62]. Given the observed correlation between *KRAS* mutation and nuclear phosphorylation of YB‐1, it may be hypothesized that in *KRAS‐*mutated cancer cells, increased YB‐1 activity may play a role in increasing sensitivity to irinotecan (a camptothecin derivative).

Most interestingly, looking at clinical studies stratifying oxaliplatin and irinotecan‐based treatment outcomes by *KRAS* mutation status, it is reported that *KRAS*‐mutated CRC responds worse to oxaliplatin‐based regimens compared to *KRAS* wildtype CRC [64, 65]. However, in the clinical treatment of CRC, currently, *KRAS* mutation status is mainly looked at to decide whether EGFR‐targeted therapies are feasible. This observation was validated by reduced EGFR expression in CRC cells following YB‐1 knockdown [66]. Thus, treatment with EGFR‐targeted antibodies (cetuximab, panitumumab) may be assumed to be more efficient in specimens with high YB‐1 expression. On the contrary, it might also be argued that high S102 YB‐1 coming from upstream activating mutations (KRAS, MEK, ERK, PI3K) may also negate the effects of EGFR targeting similar to *KRAS* mutation. It was shown that a reduction of YB‐1 phosphorylation increases the effect of cetuximab *in vitro* in CRC cells [13], which may be a promising approach in KRAS‐mutated CRC that intrinsically does not respond well to EGFR‐targeted therapies.

This may have an impact on therapy selection and implementation, *e.g*., using topoisomerase inhibitor irinotecan instead of oxaliplatin and VEGF‐based therapy instead of EGFR‐based therapy in S102 YB‐1 high specimens. For simplification, this study has demonstrated a high correspondence of nuclear S102 YB‐1 and *KRAS* mutation—thus, these therapeutically relevant assumptions may also be transferred to *KRAS* mutated cancers as well.

While the study provides insights into the subcellular localization of YB‐1, especially in relation to S102 phosphorylation, there are important limitations to consider, particularly regarding sample size, methodological constraints, and functional implications. However, by addressing these limitations in future studies—through larger sample sizes, functional assays, and dynamic imaging techniques—this research could have a substantial impact on cancer biology and the understanding of post‐translational modifications in cancer and their clinical relevance. In consequence, determining the phosphorylation status, not only the expression status, of YB‐1 in patient samples, we could show a significant correlation between *KRAS* mutation and nuclear S102 phospho‐YB‐1. In contrast to previous studies, we could prove this activated form of YB‐1 to be present in the nucleus of CRC in patients. Nuclear S102 YB‐1 was especially present in non‐lymphonodal metastasized specimens. Additionally, we found a significant correlation of FBXW7 mutation. Further research is warranted.

## 5 Conclusion

We have investigated a cohort of CRC patients regarding the phosphorylation status of YB‐1 and discriminated its subcellular localization. Although the association between *KRAS* mutation and YB‐1 phosphorylation at S102 has been investigated extensively *in vitro*, the relationship between YB‐1 phosphorylation and the subcellular localization, and *KRAS* mutation in CRC tissues *ex vivo* remained to be elucidated. To the best of our knowledge, this was the first study to investigate this. We also broadly explored whether any clinicopathological aspects are related to nuclear S102 YB‐1 and found that apart from *KRAS* mutation, *FBXW7* mutation, and lymph node metastasis are related to the phosphorylation status of YB‐1.

By determining the nuclear level of S102 YB‐1 in CRC samples, it may be possible to identify patients who are at higher risk of cancer progression. In addition, determining S102 YB‐1 in CRC samples provides valuable insights into the underlying mechanisms of YB‐1 regulation in CRC and helps to identify potential therapeutic targets and treatments for this disease. In the future, cancer phospho‐proteomics may be a promising approach to target oncogenic pathways in patients, as well as a predictive tool for therapy selection and response monitoring.

## Conflict of interest

The authors declare no conflicts of interest.

## Author contributions

MT and KL conceptualized the study. MScharpf performed the pathological evaluation of tumor tissues prior to staining. MSchaller provided funding and established the automated staining procedures. BF carried out the IHC and IF stainings. SF evaluated the histology. IB and LM performed and analyzed the next‐generation sequencing. KL and MT conducted the data analysis, with biostatistical support from GB. The manuscript was written by KL and MT, and revised by KL, SK, and MT. MT supervised the project.

## Data Availability

The pseudonymized data set that supports the findings of this study is available from the corresponding author upon reasonable request.
